# Risk factors for ophthalmologic involvement and ocular findings in patients diagnosed with fungemia in a high-complexity hospital in the city of Medellín, Colombia

**DOI:** 10.1080/07853890.2022.2107700

**Published:** 2022-08-03

**Authors:** Marcos Restrepo Arango, Juan Camilo Cadavid Usuga, Luis Fernando Velazquez Ossa, Jorge Hernando Donado Gómez, Laura Nataly Higuita Duque, Juan Pedro Neira Gomez

**Affiliations:** a Ophthalmology program, School of Health Sciences, School of Medicine, Universidad Pontificia Bolivariana, Medellín, Colombia; b Ophthalmology Department, Hospital Pablo Tobón Uribe, Medellín, Colombia; c Investigation and Epidemiology Department, Universidad Pontificia Bolivariana and Hospital Pablo Tobón Uribe, Medellín, Colombia

**Keywords:** Fungemia, retina, candida, Fundus Oculi

## Abstract

**Purpose:**

To describe the demographic clinical characteristics and to identify the risk factors of patients diagnosed with fungemia and secondary intraocular involvement.

**Methods:**

Retrospective cohort of 97 patients diagnosed with fungemia and with or without involvement of the posterior segment. Demographic, clinical and ophthalmological variables were identified to establish the risk of retinal seeding.

**Results:**

An incidence of ocular involvement of 22.68% was obtained and no clear risk factor was found for subsequent showings in patients with fungemia. A risk trend was only found in patients with diabetes with an OR: 2.85; CI 95%: (0.80–10.12) and history of HIV with an OR: 2.29 CI95%: (0.85–6.12).

**Conclusions:**

In this first cohort carried out in Colombia according to our search, findings were obtained that agree with those of other authors worldwide, where there is no evidence of a decrease in incidence compared with older studies and the absence of risk factors for the compromise of the posterior pole in patients with fungemia.

**KEY MESSAGES**
- Systematic fundus evaluation by an ophthalmologist in patients with candidaemia is a recommended practice based on low-quality evidence.
- The identification of real risk factors for retinal compromise in fungemia would allow us to be more selective with the population to be evaluated.
- Fungemia generally occurs in critically ill patients, where access and availability of ophthalmology evaluation are a resource that is not always available.

## Introduction

Fungemia is defined as the presence of fungi in blood, most commonly caused by yeasts of the *Candida* genus. Its early detection is a diagnostic and prognostic priority, since it is associated with high mortality, which varies between 10% and 30% depending on the type of patient, the source of the infection and the initial management [1].

In recent years, various techniques for early diagnosis have been developed; these are based on the detection of antigens, antibodies and genetic material. However, the method chosen for confirmation is still blood culture, a method that allows the identification of the etiologic agent and the study of in vitro sensitivity to guide treatment [2].

Signs and symptoms are non-specific and sometimes cannot even be differentiated from bacteraemia; however, disseminated infections derived from fungi can generate retinal seeding by haematogenous route, producing ocular manifestations such as chorioretinitis or endophthalmitis, especially by *Candida* species, and thus increasing the risk of potential vision loss [3,4].

According to studies previously conducted on patients with fungal infections by blood culture, the cumulative incidence of ophthalmologic manifestations is usually below 5%. The incidence rate of fungal chorioretinitis varies from 2% to 9% and endophthalmitis averages 1%; however, the incidence rate of endophthalmitis often varies between studies because inconsistent definitions of endophthalmitis are used [5].

Given the risk that fungal retinal seeding represents for the visual outcomes of patients, the Infectious Diseases Society of America (IDSA) and European experts recommend that patients undergo at least a fundus examination, ideally performed by an ophthalmologist, under dilation [6]. However, the most recent studies report incidences of 0.05% to 0.4% being associated in almost all cases with factors such as HIV, lymphoma or leukaemia, diabetes mellitus or prolonged hospitalization in critical care. Such conditions increase the risk of experiencing the involvement despite having controlled management with systemic antifungals, which for years have shown good efficacy. Therefore, the need for ophthalmologic assessment is increasingly under discussion. In turn, the disparity of definitions of posterior pole involvement and endophthalmitis limits the homogenization of results and determination of the actual risk [7].

It is important to highlight that the current IDSA guidelines recommend ophthalmologic examination for all patients diagnosed with candidemia as well as for non-neutropenic patients to be taken the first week of diagnosis. For neutropenic patients, it should be one week after neutrophil levels have recovered. In addition, for Candida endophthalmitis and chorioretinitis, the guidelines suggest intravenous and intravitreal management for at least four to six weeks, depending on fundus follow-up and absence of lesions, with a low level of evidence [6]. In addition, in the latest recommendations published by the American Academy of Ophthalmology, a routine ophthalmological consultation is not recommended after laboratory findings of systemic septicaemia due to *Candida* [8].

This is a retrospective cohort study which aims at characterising patients diagnosed with fungemia, determining the prevalence of posterior pole involvement and exploring the risk factors associated with this involvement.

## Methodology

### Study design and population

This is an observational retrospective cohort study which included patients admitted to the Pablo Tobón Uribe Hospital in Medellín, Colombia – a fourth level of complexity institution, diagnosed with fungemia and evaluated by the ophthalmology service during seven years, between 2013 and 2020. Patients with incomplete information in their medical history and who did not authorise the use of their data were excluded from the study. The only inclusion criteria considered was that the patients were both diagnosed with fungemia and had an evaluation by ophthalmology to rule out ocular involvement. This report follows the requirements proposed by STROBE [9].

The identification of study participants was performed by filtering the histories with International Classification of Diseases version 10 (ICD-10) codes B37.9 (candidiasis, unspecified) and B49 (mycoses, unspecified) between 2013 and 2020.

The variables measured were: socio-demographic characteristics of patients (age, sex, place of residence, and occupation); risk factors for fungal infection (diabetes, steroid use, human immunodeficiency virus infection, and history of neoplasia); ophthalmologic examination findings (visual acuity, presence of chorioretinitis, endophthalmitis or non-specific findings). Endophthalmitis was defined as the infection of two or more ocular segments, where if the cornea is one of them, there must be another additional segment involved; type of fungus isolated in blood culture and antibiotics used for treatment intravenously; and other clinical characteristics at the time of diagnosis (ICU hospitalization, shock and neutrophil count).

### Data collection and processing

The information was collected through a collection form in the REDCap database, previously tested. To guarantee the quality of the information, 10% of the histories were reviewed in duplicate; for categorical variables, the kappa concordance coefficient was evaluated, whereas for continuous variables was the intraclass ratio coefficient.

### Statistical analysis

In the descriptive analysis for qualitative variables, absolute and relative frequencies were calculated. Quantitative variables are presented, according to their distribution by the Shapiro–Wilk test, as mean and standard deviation or median and interquartile range. Bivariate analysis was performed between the outcome of posterior pole ocular involvement and no involvement, with dichotomous categorical independent variables by OR, with a 95% confidence interval and *p* value, with a significance level (alpha value) of 0.05.

A sample size of 126 patients was estimated, with expected prevalence parameter of ophthalmologic manifestations of 9%, 95% confidence level and a design effect of 1.0. Sampling was performed by a simple random method of patients who met the inclusion criteria. Analyses were performed in the statistical package Epidat version 4.2 [10].

### Ethical considerations

The researchers adhered to the Declaration of Helsinki, version 2013, and the project was previously approved by the ethics committee of the participating institution. Informed consent was not required because it was a non-risk research.

## Results

### Clinical-demographic characteristics

Epidemiologic and clinical data from 97 patients were collected during the selected period. Table 1 shows the clinical-epidemiologic characteristics of the patients, divided between those who did and did not experience ocular involvement associated with the diagnosis of fungemia. A total of 56.7% (55/97) were men. The mean age for those who had ocular involvement was 50 ± 16.81 years and 35.8 ± 25.62 years for those who did not.

**Table 1.** Sociodemographic and clinical characteristics of 97 patients with ocular fungemia evaluated by an ophthalmologist.

	With ocular posterior pole involvement	Without ocular posterior pole involvement
Characteristic	(*n* = 22)	(*n* = 75)
Age		
Age in years, mean (SD)	50.0 (16.81)	35.8 (25.62)
Age in years, median (IQR)	54.5 (34.25-64.75)	35 (8-56)
Older than 50 years, *n* (%)	12 (54.55)	31 (41.33)
Older than 65 years, *n* (%)	5 (22.73)	17 (22.67)
Sex No. (%)		
Female	8 (36.36)	34 (45.33)
Male	14 (63.64)	41 (54.67)
Immunocompromised state *n* (%)		
Solid organ cancer	4 (18.18)	11 (14.66)
Haematologic cancer	6 (27.27)	25 (33.33)
HIV	10 (45.45)	20 (26.66)
Diabetes	5 (22.73)	7 (9.33)
Other characteristics *n* (%)		
Steroid use	2 (9.09)	11 (14.67)
ICU stay	12 (54.55)	45 (60.00)
Shock	12 (54.55)	32 (42.67)
Neutrophils		
Mean (SD)	6.144.09 (6.192.13)	6.985.5 (5.850.6)
Median (IQR)	4.057 (975-10.966)	5.338 (2.550-10.650)
Less than 1500, No (%)	6 (27.27)	13 (17.33)
Less than 1000, No (%)	6 (27.27)	11 (14.67)
Less than 500, No (%)	3 (13.64)	8 (10.67)
Deceased *n* (%)	10 (45.45)	12 (16)
Isolated fungus *n* (%)		
*Candida albicans*	79 (81.44)	
*Candida tropicalis*	6 (6.18)	
*Candida parapsilosis*	3 (3.09)	
Histoplasma capsulatum	2 (2.06)	
*Candida auris*	1 (1.03)	
Aspergillus	1 (1.03)	
Cryptococcus	1 (1.03)	
No data	4 (4.12)	
Intravenous Treatment *n* (%)		
Fluconazole	49 (50.52)	
Voriconazole	9 (9.28)	
Caspofungin	38 (39.18)	
Amphotericin B	21 (21.65)	
Itraconazole	2 (2.06)	
Ophthalmologic findings *n* (%)		
Chorioretinitis	6 (6.19)	
Endophthalmitis	1 (1.03)	
Ischemic areas	0 (0)	
Haemorrhages	10 (10.31)	
White spots	17 (17.53)	
None	74 (76.28)	

The history of solid organ cancer was 36.36% (4/22), haematologic cancer 27.27% (6/22), HIV 45.45% (10/22) and of diabetes 22.73% (5/22) in patients with ocular involvement. In the highest percentage of patients, that is, 91.75% (89/97), some type of *Candida* fungus was isolated; and patients who were given intravenous fluconazole or caspofungin were 50.52% (49/97) and 39.18% (38/97), respectively.

The only inclusion criteria considered for the study were patients with a diagnosis of fungemia who had been evaluated by an ophthalmologist to rule out ophthalmologic involvement. Of the patients evaluated, 58.76% (57/97) were in ICU at the time of evaluation, 45.36% (44/97) were in shock and 13.40% (13/97) had been on steroids at that time due to illness before evaluation. Of the total number of patients evaluated, 42.27% (41/97) died during the same hospitalization period as that of the ophthalmologic examination. The percentage of deceased within the ocular involvement group was 24.39% (10/22) vs. 21.43% (12/97) of those without ocular involvement.

Data quality was assessed with a duplicate review of 10% of the medical histories. The concordance coefficient (Kappa) applied for categorical variables was 0.74 CI (0.64–0.85) *p*-value .000 and the interclass correlation coefficient (ICC) for continuous variables was 0.87 CI 95% (0.77–0.93).

### Ophthalmologic characteristics and associated risk factors

Visual acuity in these patients was difficult to collect, since in 69% (71/97) of the cases it was not evaluated. This was due to the fact that more than half of the patients were in the ICU under sedation and another percentage could not be transferred to the doctor’s office for evaluation. Hence, these data are not shown independently. However, the most frequent value, with 11% (10/97) was visual acuity of 20/30. Table 1 shows the different findings in the fundus of the patients, being white spots (cotton wool spots) and haemorrhages the most frequently found.

Ocular involvement was present in 22 of 97 patients, representing 22.68% with a 95% CI of (14.7–30.30). Among the variables evaluated as possible risk factors, diabetes had an OR of 2.85 (95% CI: 0.80–10.2), HIV diagnosis had an OR of 2.29 (95% CI: 0.85–6.12), neutropenia less than 1000 and less than 500 (absolute number) showed an OR of 1.97 (0.63–6.17) and 1.20 (0.29-4.99). Table 2 shows all the variables with their point estimates and respective confidence intervals.

**Table 2.** Risk factors associated with ocular involvement by fungemia.

**Variables** **N (%)**		**With ocular involvement** **(*n* = 22)**	**Without ocular involvement** **(*n* = 75)**	OR CI 95%	*p* Value
**Male Sex**	**Yes**	14 (63.64)	41 (54.67)	1.45 (0.54-3.86)	.61
	**No**	8 (36.36)	34 (45.33)		
**Older than 65 years (*n* = 97)**	**Yes**	5 (22.73)	17 (22.67)	1.00 (0.32-3.11)	1.00
	**No**	17 (77.27)	58 (77.33)		
**Older than 50 years (*n* = 97)**	**Yes**	12 (54.55)	31 (41.33)	1.70 (0.65-4.43)	.39
	**No**	10 (45.45)	44 (58.67)		
**Diabetes** **(*n* = 97)**	**Yes**	5 (22.73)	7 (9.33)	2.85 (0.80-10.12)	.19
	**No**	17 (77.27)	68 (90.67)		
**HIV** **(*n* = 97)**	**Yes**	10 (45.55)	20 (26.66)	2.29 (0.85 − 6.12)	.09
	**No**	12 (54.55)	55 (73.33)		
**Solid tumor** **(*n* = 97)**	**Yes**	4 (18.18)	11 (14.66)	1.29 (0.36 − 4.55)	.68
	**No**	18 (81.81)	64 (85.33)		
**Haematologic tumor** **(*n* = 97)**	**Yes**	6 (27.27)	25 (33.33)	0.75 (0.26-2.15)	.59
	**No**	16 (72.72)	50 (66.66)		
**PMNs < 1500** **(*n* = 91)**	**Yes**	6 (27.27)	13 (18.84)	1.61 (0.52-4.92)	.58
	**No**	16 (72.73)	56(81.16)		
**PMNs < 1000** **(*n* = 91)**	**Yes**	6 (27.27)	11 (15.94)	1.97 (0.63-6.17)	.38
	**No**	16 (72.73)	58 (84.06)		
**PMNs < 500** **(*n* = 91)**	**Yes**	3 (13.64)	8 (11.59)	1.20 (0.29-4.99)	1.00
	**No**	19 (86.36)	61 (88.41)		
**Steroid** **(*n* = 97)**	**Yes**	2 (9.09)	11 (14.67)	0.58 (0.11 − 2.84)	.74
	**No**	20 (90.91)	64 (85.33)		
**ICU stay (*n* = 97)**	**Yes**	12 (54.55)	45 (60.00)	0.80 (0.30 − 2.08)	.83
	**No**	10 (45.45)	30 (40.00)		
**Shock** **(*n* = 97)**	**Yes**	12 (54.55)	32 (42.67)	1.61 (0.62-4.19)	.45
	**No**	10 (45.45)	43 (57.33)		
**Deceased** **(*n* = 97)**	**Yes**	10 (24.39)	12 (21.43)	1.18 (0.45-3.07)	.92
	**No**	31 (75.61)	44 (78.57)		
**Older than 50 years (*n* = 97)**	**Yes**	12 (54.55)	31 (41.33)	1.70 (0.65-4.43)	.39
	**No**	10 (45.45)	44 (58.67)		
**C.albicans vs. other (*n* = 97)**	**Yes**	17	5	0,71 (0.22–2,28)	.56
	**No**	62	13		

## Discussion

We found an incidence of ocular involvement in fungemia of 22.68% (22/97), high frequency of intensive care unit stay (58.7%) and shock (45.3%). There was a tendency to ocular involvement in diabetic and HIV patients. Table 3 illustrates other studies that investigated similar outcomes.

**Table 3.** Additional studies determining prevalence and risk factors for ocular involvement by fungemia.

Author	Year^č^	Type of study	*n* (#)	OI*^,^^+^	Conclusion
Donahue et al. [10]	1994	Observational prospective	118	∞9%	Patients with *Candida* vs. other fungi and immunosuppressed should be evaluated^
Price et al. [11]	2017	Retrospective	95	9.5%	The study recommends assessment of patients who cannot communicate or who express vision complaints^$^
Kato et al. [12]	2018	Retrospective	174	^a^20.1%	Risk of endophthalmitis in *Candida* isolation and CVC use.
Breazzano et al. [5]	2019	Systematic Review	7412	^b^0.9%	The study questions the need for routine screening.
Son et al. [13]	2019	Retrospective	275	21.5%	The study recommends routine screening of these patients^Ç,&^
Ueda et al. [14]	2019	Retrospective	781	19.5%	The study recommends early routine screening^Ø^
Shin et al. [15]	2020	Retrospective	225	12.9%	The study suggests active search for complications, including ocular involvement^κ^

č year of publication.

*n*: number of patients assessed ophthalmology.

+Defined as endophthalmitis or retinal lesions.

^ *p* value not significant.

* OI: ocular involvement.

∞Candida chorioretinitis.

^a^ Chorioretinitis and endophthalmitis.

^b^ Endophthalmitis according to the article's own definition.

$ No risk associations.

CVC: central venous catheter.

& Greater than 72 h after initiation of treatment.

Ç: the patients were evaluated in the first two weeks after the diagnosis of candidaemia.

Ø: Based on average diagnosis at 5.0 ± 3.9 days after established candidaemia.

κ: Based on average diagnosis seven days after established candidaemia.

Vaziri et al. [11] reported in their study a total of 82.8% in the ICU when they were diagnosed with endophthalmitis and 3.3% with HIV. Rodríguez-Adrián et al. [12] studied 100% of their patients in the ICU, whereas Ueda et al. [13] only reported 17.4%, with 13.95% in shock. Kato et al. [14] had 31. 6% of their patients in an ICU at diagnosis and 19% in shock. This suggests that the patients in our sample were possibly sicker compared with the studies that were reviewed.

It is difficult to determine the real prevalence of posterior pole involvement in patients with fungemia due to the heterogeneity of criteria and retinal lesions included in the different studies. Although there may be retinal seeding, they may be due to the patient's own multisystemic involvement. The IDSA recommends, as part of the optimal management of the patient with fungemia, the ophthalmologic examination under dilation by an ophthalmology specialist to rule out intraocular involvement. However, the emergence of new antifungals with good ocular penetrance, reports of successful cases of intravenous management as monotherapy [16], the non-inferiority of echinocandins in reducing the rate of ocular involvement [17] and the decrease in the incidence of intraocular involvement over time call into question the need for this evaluation systematically. This is supported by the recommendation against routine ophthalmological examination in these patients suggested by the American Academy of Ophthalmology [8].

The rate of ocular involvement by fungemia has been described in several studies, ranging from 0.9% to 40% [5], depending on whether endophthalmitis or chorioretinitis is evaluated, with endophthalmitis showing the lowest rates of involvement. For our study the rate was 22.68%, very similar to the findings found by Son et al. [18], 21.5%. The difference between studies was that Son’s only took into account *Candida* fungemia, whereas ours considered any type of fungus. Ueda et al. [13] found a percentage of *Candida* infection of 77.9%, similar to ours, which was 81.4% for their species C. *albicans* and higher for all *Candida* species with 91.7%. In some studies, such as the one by Rodríguez-Adrián et al. [12], they report that at least 87% of patients with ocular involvement had at least one condition other than fungemia that could explain the fundus findings, such as diabetes, HIV infection, hypertension and leukaemia. It is interesting to clarify that although in our study the majority of patients with ocular involvement had positive cultures for C. albicans, it did not represent a statistically significant risk. Similarly, our study found that only six patients had clear chorioretinitis and one of them endophthalmitis, which corresponds to 31% of patients with ocular involvement, whereas the other 69% represents non-specific findings that may be due to the previously described conditions also presented in our population. This means that excluding non-specific findings, the incidence of posterior pole involvement for our population is 7.21%.

Neutropenia, immunosuppression status and the presence of central venous catheter (CVC) have been suggested as risk factors for the development of ocular involvement; however, the statistical support is not solid. In this study, neutropenia less than 1000 or less than 500 represented a risk factor with an OR of 1.97 and 1.20 respectively, but with an imprecise 95% CI and non-significant *p* value. Solid and haematologic organ neoplasia tended to be a risk factor, but without statistical significance, which could be explained by the sample size. Likewise, Son et al. [18,19] reported neutropenia as an associated factor without statistical significance; and HIV or a history of neoplasia did not represent a risk factor, just like in our study. The difference is that in our study, HIV shows a risk trend that could be positive with an increase in the sample size.

As in our work, Shin et al. [20] and Ueda et al. [13] do not support the presence of diabetes, previous steroid use or the presence of shock as a risk factor. It is important to consider that Shin et al. [20] evaluated the global risk of endophthalmitis, endocarditis and osteomyelitis and that only 29% of patients underwent fundus examination. They identified an overall rate of candidaemia complications of 4.4% (34/765), of which 3.8% had endophthalmitis. It is important to clarify that they found that 30.3% of the patients with some complication of candidaemia had diabetes, but they did not specify how many had endophthalmitis. In their conclusions they do not identify diabetes as a risk factor for complications. Same as our results. Ueda et al. [13] in turn divided intraocular involvement between presence of lesions with or without macular involvement and endophthalmitis, calculating for each one the risk; and still not finding any.

Finally, in a meta-analysis by Breazzano et al. [5], based on 38 studies and 7412 patients; individually, the studies showed incidences ranging from 0% to 52%, with an average of 17.9% before 1994 and 1.2% after 1994. They conclude that routine ophthalmologic examination in visually asymptomatic patients diagnosed with candidaemia is not necessary, even in patients who cannot verbalize, and that cases should be evaluated independently.

The strength of this study is that it is the first work of this nature carried out in Colombia, being performed in a high-complexity institution, where critical patients are treated in an integral manner and referral to other specialists is common to rule out intraocular involvement due to fungemia. In addition, possible risk variables described in the literature were taken into account, the level of neutropenia was divided to be more specific, and all patients with ophthalmologic examination, both with positive and negative findings, were considered to establish the risk.

As a weakness, this is a study of retrospective and single-centre nature, so it would be of great importance to perform a prospective multicentre study in Colombia to support these findings. Secondly, the non-specific fundus findings may be explained by the stay in the ICU, the state of shock and the presence of decompensated systemic diseases; however, this is difficult to differentiate and there will always be this uncertainty, so, in that sense, our findings are not weakened. It should also be considered that despite having reviewed seven years of medical histories, the sample was slightly smaller than expected than the one calculated for the study. This study is applicable to Colombian patients who are in an institution with a high level of complexity within the country's health insurance system. Since there was no random sampling, representativeness may be limited.

This being the first study conducted in Colombia, according to our search, has important implications on the daily practice of response to referral to rule out intraocular involvement in patients with fungemia. This is supported by similar results regarding the low incidence of intraocular involvement and the absence of identifiable risk factors for developing endophthalmitis or chorioretinitis, in addition to the history of HIV. Therefore, based on the protocol for fundus assessment by an ophthalmologist – in a social and economic context where access to this specialty is not generalized and it is uncommon and expensive, it is suggested that the need for assessment be contemporised and individualized for each case independently.

## Conclusions

In summary, although the risk of intraocular involvement in patients with fungemia, especially by *Candida* species, is described in the literature, our study, just like others worldwide, presents the real low incidence and the non-identification of risk factors that justify routine screening in all patients, except for a history of HIV and diabetes which tend to risk. This, together with other studies, supports the possibility of updating the management guidelines for this type of patients when larger, randomized, multicentre studies are conducted.

## Data Availability

Data supporting the findings of this study are available upon request from the corresponding author, M.R. The data is not publicly available due to restrictions because it contains information that could compromise the privacy of research participants.
